# A survey of beetles (Coleoptera) from the tundra surrounding the Nunalleq archaeological site, Quinhagak, southwestern Alaska

**DOI:** 10.3897/BDJ.6.e22788

**Published:** 2018-03-02

**Authors:** Véronique Forbes, Derek Sikes

**Affiliations:** 1 Université de Bordeaux, Bordeaux, France; 2 University of Aberdeen, Aberdeen, United Kingdom; 3 Memorial University of Newfoundland, St. John's, Canada; 4 University of Alaska Museum, Fairbanks, United States of America

**Keywords:** Coleoptera, Archaeology, Insect subfossils, Alaska, Yup’ik

## Abstract

This paper presents the results of a survey of beetles conducted in the vicinity of the archaeological site of Nunalleq, a pre-contact (16^th^-17^th^ century AD) indigenous forager settlement located near the modern Yup’ik village of Quinhagak, in the Yukon-Kuskokwim delta, southwestern Alaska. Records and habitat data are reported for 74 beetle taxa collected in tundra, riparian, aquatic and anthropogenic environments from a region of Alaska that has been poorly studied by entomologists. This includes the first mainland Alaskan record for the byrrhid *Simplocaria metallica* (Sturm). Beyond improving our knowledge of the local beetle fauna’s diversity and ecology, this survey provides the basis for comparisons between modern and sub-fossil beetle assemblages from Nunalleq and Quinhagak.

## Introduction

Until recently, the arthropod fauna of Alaska has been comparatively less well studied than that of other states and provinces of Canada and the USA. In part this is caused by the fact that some regions are particularly difficult to access due to their topography, hydrology or remoteness to urban agglomerations. This is the case of the Yukon-Kuskokwim (Y-K) delta, a flat, treeless area of south-western Alaska where the tundra environment is dissected by numerous rivers, streams, lakes and ponds and underlain by discontinuous permafrost. Travel by motorised vehicle is impractical over most of the delta’s expanse, making small boats and planes the most reliable transport means within the area. Karl Lindroth and Georges E. Ball are two of the few entomologists known to have visited the region, when, as part of Lindroth’s seminal study of the ground beetles of Canada and Alaska (Lindroth 1961, Lindroth 1963b, Lindroth 1966, Lindroth 1968, Lindroth 1969a, Lindroth 1969b), they conducted a survey of the carabids of the western Alaskan tundra. Little entomological work has been done in the area since this date (although see Hayford et al. 2014 for a survey of chironomids (Diptera: Chironomidae) from the region).

From 2013, one of the team (VF) became engaged in a scientific, community and heritage project involving the excavation of Nunalleq, a pre-contact Thule-era (16^th^-17^th^ century AD) site located on the Bering Coast of the Yukon-Kuskokwim delta, approximately 20 km south of the Yup’ik village of Quinhagak. One of the objectives of the project was to reconstruct past climatic conditions and human environment-interactions on the basis of ecological information derived from beetle remains preserved in the archaeology (Forbes et al. 2015). Similar palaeo-environmental reconstructions based on sub-fossil insect fauna have been conducted in Alaska, although on older deposits, most of which were not directly connected to human occupations (e.g. Bigelow et al. 2014,Elias 1992, Elias 2000, Elias 2001, Elias and Matthews 2002, Elias and Short 1992, Elias et al. 1992, Elias et al. 1996, Elias et al. 1997, Elias et al. 1999, Elias et al. 2000, Matthews et al. 2003, Reyes et al. 2010, Reyes et al. 2011, Wilson and Elias 1986, Wooller et al. 2011). Sound knowledge of the insect fauna of the study locale is required to both successfully identify disarticulated sub-fossil remains and derive ecological information from them (Elias 2010, Forbes et al. 2016). For this reason, it was decided to conduct a small-scale entomological survey concurrently with the archaeological excavations at Nunalleq. The objective of the survey, beyond familiarisation with the local fauna, was to obtain a sample of the modern beetle fauna from the coastal tundra and anthropogenic habitats with which the beetle sub-fossils from Nunalleq could be compared. This paper reports the list of taxa obtained and discusses their significance for palaeo-entomological and archaeo-entomological research.

## Material and methods

**Figure 1.**
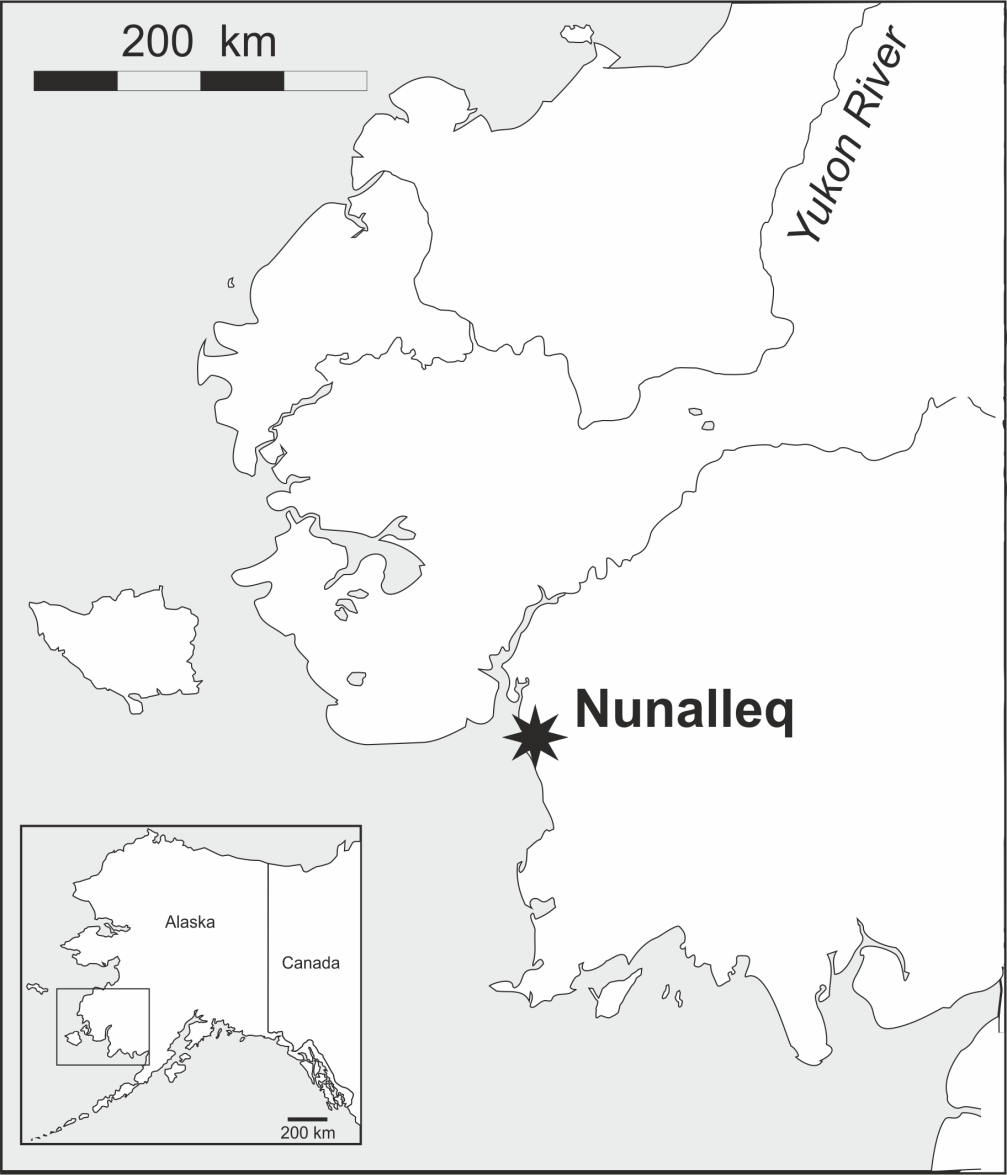
Map of the Yukon-Kuskokwim Delta region of Alaska, showing the location of the Nunalleq archaeological site (image by P. Ledger).

Fieldwork was conducted during two consecutive field seasons within a 5 km radius of the Nunalleq archaeological site (59°42.559' N 161°53.510' W WGS84, Fig. 1). The first season ran from the 28^th^ of July to the 28^th^ of August 2014 and the second, from the 2^nd^ of July to the 10^th^ of August 2015. In order to obtain a sample as representative as possible of the local beetle fauna’s diversity (within the constraint imposed by logistics and timing), different techniques were selected (Table 1). Pitfall traps were used to capture beetles crawling on the tundra, the seashore and disturbed ground around the archaeological excavation. They were made of plastic cups (7 cm diameter and 9 cm deep) half-filled with seawater, to which a few drops of dishwashing liquid were added (Fig. 2a). To capture flying beetles, flight interception traps were used, made up of a black mosquito mesh (1.2 m high and 1.4 m wide) stretched vertically between two posts. A set of plastic food containers half-filled with seawater was placed beneath the mesh to collect the insects (Fig. 2b). The pitfall and interception traps were emptied twice per week. Additional sampling techniques included beating vegetation, dipping and sifting and/or separation using a mini-Winkler extractor (Fig. 2c) and hand collecting.

Identifications of the beetle taxa were achieved through anatomical comparisons with specimens from the University of Alaska Museum Insect Collection (UAM) in Fairbanks, the Canadian Collection of Insects, Arachnids and Nematodes in Ottawa (CNC) and the Laurentian Forestry Centre’s René-Martineau Insectarium in Quebec City (LFC). For some specimens, such as those belonging to the Staphylinidae subfamilies Aleocharinae, Omaliinae and Staphyliniinae, as well as the Pterostichus subgenus Cryobius Chaudoir, this was facilitated by microdissections to allow observation of the genitalia. Identifications were aided by consultation of identification keys and descriptions in entomology publications (Anderson and Peck 1985, Arnett and Thomas 2001, Arnett et al. 2002, Askevold 1991, Bousquet 1990, Bousquet 2010, Bright 1987, Buchanan 1927, Campbell 1978, Campbell 1984, Campbell 1991, Campbell 1983, Campbell 1982, Campbell 1973, Casey 1884, Downie and Arnett 1994, Downie and Arnett 1996, Gusarov 2004, Hatch 1971, Johnson 1991, Larson et al. 2000, Lindroth 1961, Lindroth 1963b, Lindroth 1966, Lindroth 1968, Lindroth 1969a, Lindroth 1969b, Klimaszewski et al. 2011, Klimaszewski et al. 2008, Lohse et al. 1990, O'Brien 1970, Ratcliffe 1996, Shavrin 2016, Smetana 1971, Wells 1991). Many specimens were identified, or had their identification confirmed, by taxonomic specialists. The taxonomic classification of Bousquet et al. (2013) was used.

Information regarding the ecology of individual taxa was compiled from habitat records and descriptions in the literature (Anderson and Peck 1985, Arnett and Thomas 2001, Arnett et al. 2002, Askevold 1991, Ball 1966, Bousquet 1990, Bousquet et al. 2013, Campbell 1973, Campbell 1978, Campbell 1982, Campbell 1983, Campbell 1984, Campbell 1991, Collet 2008, Elias 1992, Erwin 2007, Gordon 1985, Gusarov 2004, Hicks 1959, Horn 1880, Klimaszewski et al. 2011, Klimaszewski et al. 2008, Klimaszewski et al. 2016, Larochelle and Larivière 2003, Larson et al. 2000, Lindroth 1961, Lindroth 1963b, Lindroth 1966, Lindroth 1968, Lohse et al. 1990, Majka and Langor 2008, Majka and Langor 2011, Matthews 1983, O'Brien 1970, Ratcliffe 1996, Shavrin 2016, Sikes et al. 2016, Smetana 1971). This was then used to classify these taxa into broad habitat categories, as is customary in archaeo-entomological studies (Buckland 2009, Kenward 2009). The authors also queried the distribution data available for each of the taxa represented in the dataset in an attempt to identify potential first records for the Y-K delta region. This was achieved by undertaking a search in the Arctos database (arctos.database.museum), using the spatial query tool set to the aforementioned region as a search criterion. For carabid, dytiscid and staphylinid species,this process was complemented by examining locality records in works published by Lindroth (1961), Lindroth (1963a), Lindroth (1963b), Lindroth (1966), Lindroth (1968), Lindroth (1969a), Lindroth (1969b), Larson et al. (2000), Campbell (1973), Campbell (1978), Campbell (1982), Campbell (1983), Campbell (1984), Campbell (1991) and Smetana (1971).

## Data resources

Vouchers specimens were donated to UAM, CNC and LFC and the remaining specimens are currently in the care of the first author. Data for specimens that were donated to UAM (accession: UAM-2014.20-Forbes-Ento) can be accessed through the Arctos database using the following link  http://arctos.database.museum/saved/QuinhagakColeoptera. The full dataset is archived online and can be accessed at: doi.org/10.6084/m9.figshare.5630296.v1.

## Result & Discussion

**Figure 5.**
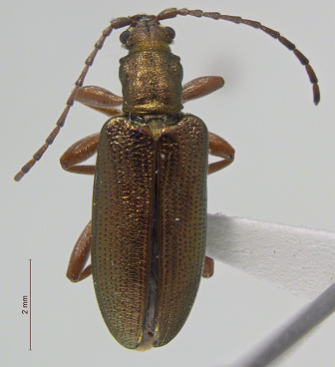
Photographs of some of the beetle species identified from Quingahak (continued). *Plateumaris flavipes*

This survey recovered a total of 500 beetle specimens belonging to 74 different taxa and spanning 15 families (Suppl. material 1, Figs 3, 4, 5). In total, 61 of the 74 taxa collected were successfully identified to species level. Of those, 50 are Holarctic in distribution, with the remaining consisting of Nearctic species. One species, *Simplocaria metallica* (Sturm), is of Holarctic distribution but considered adventive in North America (Bousquet et al. 2013), where it has been recorded from Labrador, Newfoundland, Atlantic Canada and Greenland (Majka and Langor 2011). In Alaska, it has previously been collected from St. Matthew Islands (Sikes et al. 2016), but the record from Quinhagak is the first for mainland Alaska.

Thirty-four of the identified taxa may be first records for the Y-K delta region. Many of these were collected in regions adjacent to the Y-K delta (e.g. the Seward, Alaska and Kenai peninsulas as well as central Alaska). This applies to *Notiophilus borealis* Harris, *Elaphrus lapponicus* Gyllenhal, *Hydroporus lapponum* Gyllenhal, *H. morio* Aubé, *H. striola* (Gyllenhal), *Acidota quadrata* (Zetterstedt) and Eucnecosum cf. tenue LeConte). They have probably been established in the Y-K delta for a long time, but perhaps were never collected before simply due to geographical sampling bias.

This survey also produced several records of *Amara alpina* (Paykull). This species is generally considered an indicator of cold climates in palaeo-entomological studies (Elias 2010). Seventeen specimens were collected at Quinhagak, which is characterised by a subarctic coastal climate. The nearest observational data comes from Bethel Airport (approximately 115 km northeast of Quinhagak) where mean winter (January) and summer (July) temperatures for the period 1987-2016 are -14.2 and 13.4°C respectively (NOAA 2017). Additional records in Alaska include those from St. George Island, ca. 50 km off the coast of mainland Alaska and Round Island, just 15 km south of the Y-K delta. Several records from the coast of the Y-K delta also appear in a distribution map for the species provided in Lindroth (1963a). The subfossil record suggests that the species occupied unglaciated regions of Alaska and the Yukon throughout the Quaternary (Reiss et al. 1999). Palaeo-entomological and genetic data identify this region as the principal centre-of-origin for *A. alpina* and other arctic and subarctic species that dispersed throughout northern areas of North America following the last glacial maximum (Ashworth 1996, Schwert and Ashworth 1988, Reiss et al. 1999).

Three dytiscid specimens were identified as *Ilybius angustior* (Gyllenhal) complex. These appear to be closely related to the species *I. angustior*, a Holarctic species occuring in still water with abundant vegetation (Larson et al. 2000). However, the Quinhagak specimens differ in size and colour as well as in the shape of the male metatarsal claws and aedeagus (according to Larson, personal communication, 2016).

### Ecological grouping of taxa

**Figure 6.**
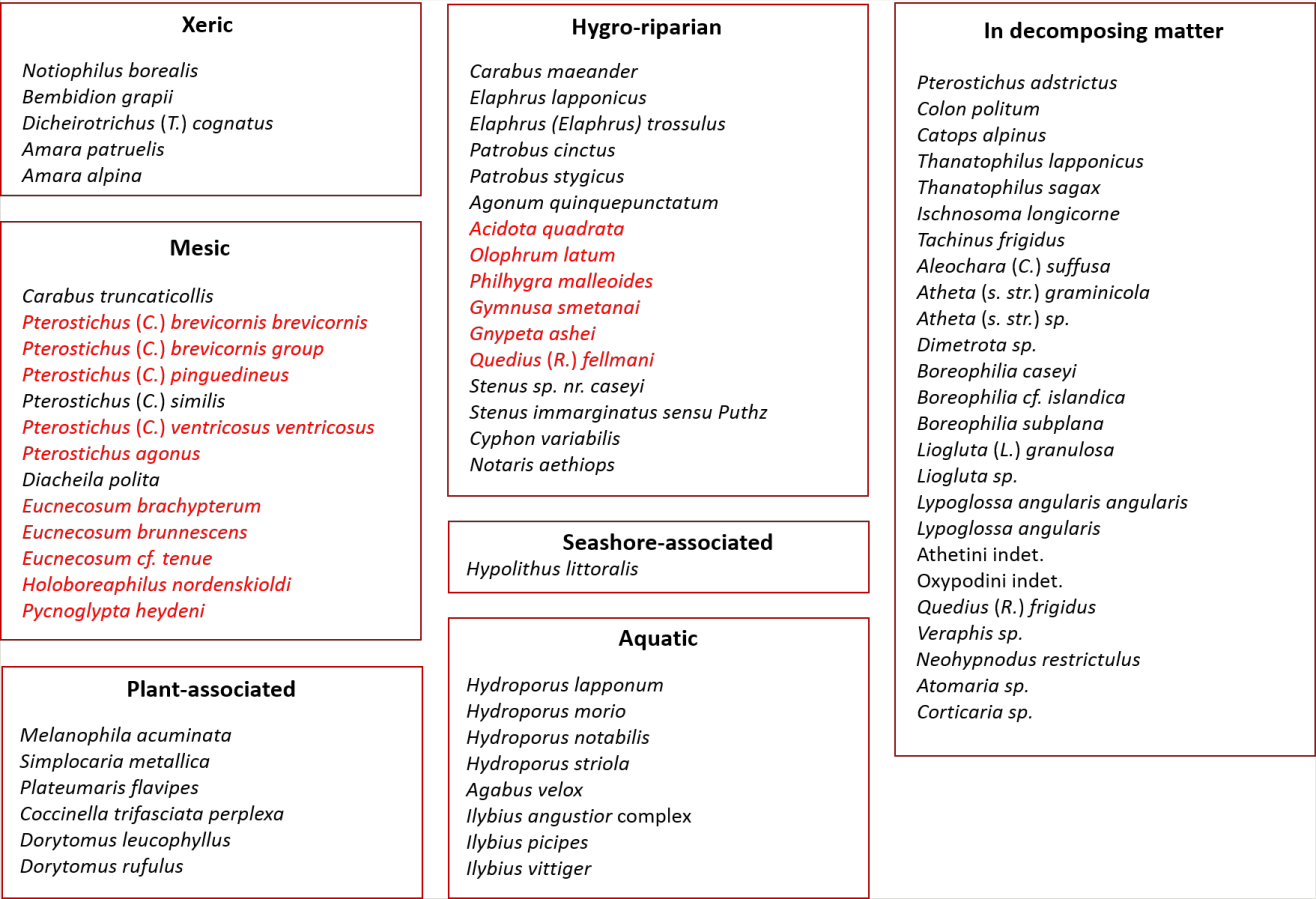
Grouping of identified taxa according to their habitat/ecology. In red font are those taxa that belong to mesic, hygrophilous and riparian environments, but that are known to be associated with microhabitats available in decaying plant matter (e.g. leaf litter, floor debris).

**Figure 7.**
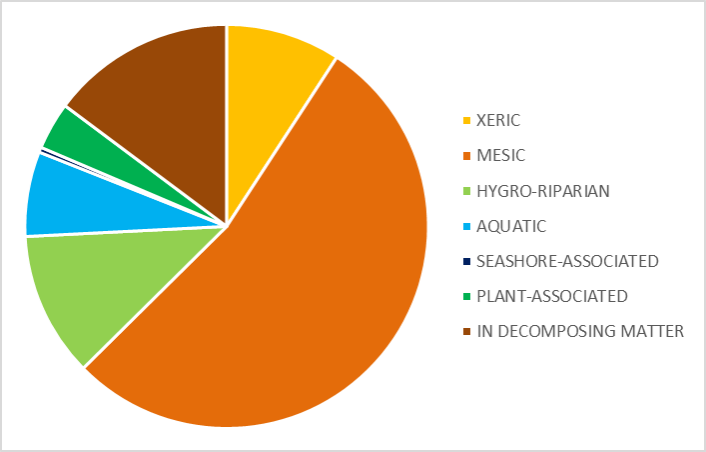
Relative proportion of each ecological group (as defined in Figure 6) represented in the sample. Percentages were calculated on the basis of the total number of Coleoptera within the sample.

Each identified taxon has been classified into an ecological group (Fig. 6). The ‘Xeric’ group contains ground beetle species that prefer dry conditions and live in the open, on ground with little to no vegetation cover. Taxa that are typical of mesic tundra habitats, which encompasses the shrub tundra but also moderately moist areas of the open tundra, have been attributed to the ‘Mesic’ group. This includes members of the subgenus Cryobius and rove beetles such as *Eucnecosum* spp. This group is dominant in this assemblage, totalling about half the total beetle specimens captured Fig. 7. Beetles preferring wet habitats and the banks of lakes or rivers were placed in the group ‘Hygro-riparian’, which includes several carabids and rove beetles, but also the scirtid *Cyphon variabilis* (Thunberg) and the brachycerid *Notaris aethiops* (Fabricius). The elaterid *Hypolithus littoralis* Eschscholtz occurs on ocean beaches and is the only species attributed to the group ‘seashore-associated’. The ‘Aquatic’ group contains the eight predacious scavengers beetle taxa identified in this study. Most aleocharines have been placed in the group ‘In decomposing matter’, alongside other rove beetles as well as mycetophagous (e.g. *Atomaria* sp. and *Corticaria* sp.) and carrion beetles (*Colon politum* Peck & Stephan, *Catops alpinus* Gyllenhal, *Thanatophilus lapponicus* (Herbst) and *T. sagax* (Mannerheim)). The click beetle *Neohypdonus restrictulus* (Mannerheim) was also placed in this group as it is believed to be omnivorous, feeding on decaying animal and plant matter (DSS, unpublished data). The ‘Plant-associated’ group includes taxa that feed directly on plants, but also the predator *Coccinella trifasciata perplexa* Mulsant, which preys on arthropods closely associated with plants (cf. Matthews 1983).

Taxa that are typical of mesic to wet tundra habitats and which occur on both sides of the Bering and Chukchi Seas, are the most represented in this survey (Fig. 7). About a third of the species identified have been recorded from other late Quaternary sites also located in south-western Alaska, both inland and just west of the Alaska Peninsula (Elias 1992). The proportion occupied by hygrophilous and riparian beetles is, however, surprisingly limited in view of the abundance of water in the area. This is no doubt in part due to the fact that sampling took place during the driest period of the year, although sampling bias probably also played a role, given that mesic tundra habitats were more easily accessible and sampled than wetter ones. Notable absentees from this study include several *Stenus* species, which are common in other subfossil assemblages of Alaska and Siberia (e.g. Elias 1992, Elias et al. 2000, Elias et al. 1996). Here, only two specimens were collected. Many *Stenus* species are riparian, hunting prey at the muddy banks of ponds or streams and are better retrieved through hand collection – by lifting rocks or streamside washing, for example. Diverse *Stenus* species probably occur around the Nunalleq archaeological site, but unfortunately, their niche(s) seem to have escaped the authors' attention. It is also interesting that the only *Bembidion* species identified in this survey is described by Lindroth as ‘not at all riparian’ (Lindroth 1963b), given that the majority of species from this genus live close to water.

Many of the taxa included in the ‘Xeric’ and ‘Mesic’ groups are typical of tundra environments, but appear to exploit niches provided by decomposing organic matter, for example rotting wood, leaf litter and flood debris (Fig. 6). This poses an interesting problem for archaeo-entomological interpretations. The most common aim of such studies is to produce high (spatial and temporal) resolution reconstructions of ecological conditions and activity areas within settlements. In this context, the importance of a species’ microhabitat preference (e.g. decaying vegetation) may outweigh that of its macrohabitat (e.g. tundra). Human settlements have been shown to generate an abundance of nutrient-rich ecological niches which is unmatched in natural situations (Forbes et al. 2014, Forbes et al. 2017). Indeed, subfossil insect faunas extracted from floors and middens on archaeological sites are typically dominated by predators and mould-feeders in decomposing vegetation, many of which are known to occupy similar niches in forest litter, mammal and bird nests and burrows or tree hollows (Kenward and Allison 1994). This is the case not only for permanent urban and rural settlements, but also for the seasonally-occupied houses of arctic and subarctic foragers, which are strongly dominated by taxa such as *Aleocharinae* indet., *Eucnecosum* spp. and *Pycnoglypta* spp. (Forbes et al. 2017). It is therefore likely that tundra species known to inhabit decomposing matter in natural situations were able to colonise the nutrient-rich niches available inside and around sod dwellings in the past. It is worth noting that this survey collected several species typical of tundra environments (e.g. *Carabus truncaticollis* Eschscholtz, *Diacheila polita* (Faldermann), Pterostichus (Cryobius) similis Mannerheim and *Pterostichus agonus* Horn) in synanthropic situations. Future archaeo-entomological analyses at Nunalleq will hopefully clarify the significance of these beetles in the reconstruction of past foraging lifeways and ecology.

## Supplementary Material

Supplementary material 1 List of beetle taxa identified at Quinhagak, with a summary of their habitats/ecology Data type: Occurences and habitat data
Brief description: List of beetle taxa identified at Nunalleq, with a summary of their habitats/ecology. Ticked (✓) boxes indicate habitat records collected as part of this survey, those marked with an ‘x’ represent habitat records identified from the literature.
Ecology and habitat abbreviations: BG = bare ground; BP = bogs, peaty soils/wet meadows; CA = carrion; CG = coastal grassland; DS = disturbed/synanthropic habitats; DT = dry tundra/heath; DU = dung; DV = decaying vegetal matter; F = fungi; H = hygrophilous; MO = moss; MT = mesic tundra; NB = nests and burrows; O = open ground; R= riparian; SB= seashore, beach; SW= standing water; TV = thin/sparse vegetation; WB = wood/bark; WL = forest, woodland, scrubs; WT = wet tundra; X = xeric habitats.
The ‘ID’ column designates the determiner for each taxa (PB = Patrice Bouchard; YB = Yves Bousquet; AD = Anthony Davies; HD = Hume Douglas; VF = Véronique Forbes; JK = Jan Klimaszewski; DL = Dave Larson; DS = Derek Sikes; AS = Aleš Smetana; MKT = Margaret K. Thayer).
The ‘DIS’ column details each taxon’s global distribution (Ho = Holarctic; Ne = Nearctic; Ad = adventive to North America).
File: oo_171276.xlsx Veronique Forbes

## Figures and Tables

**Figure 2a.**
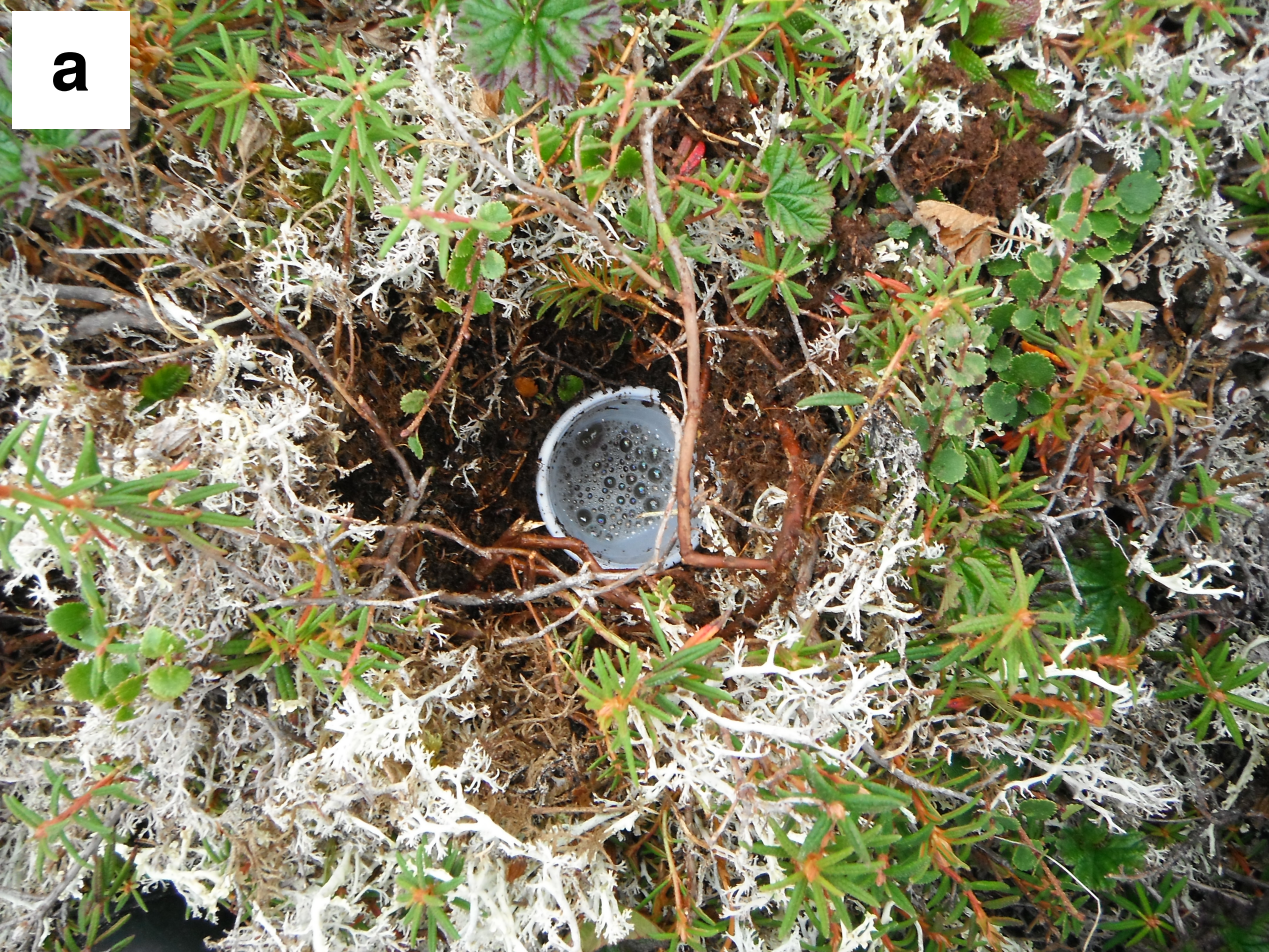
pitfall trap

**Figure 2b.**
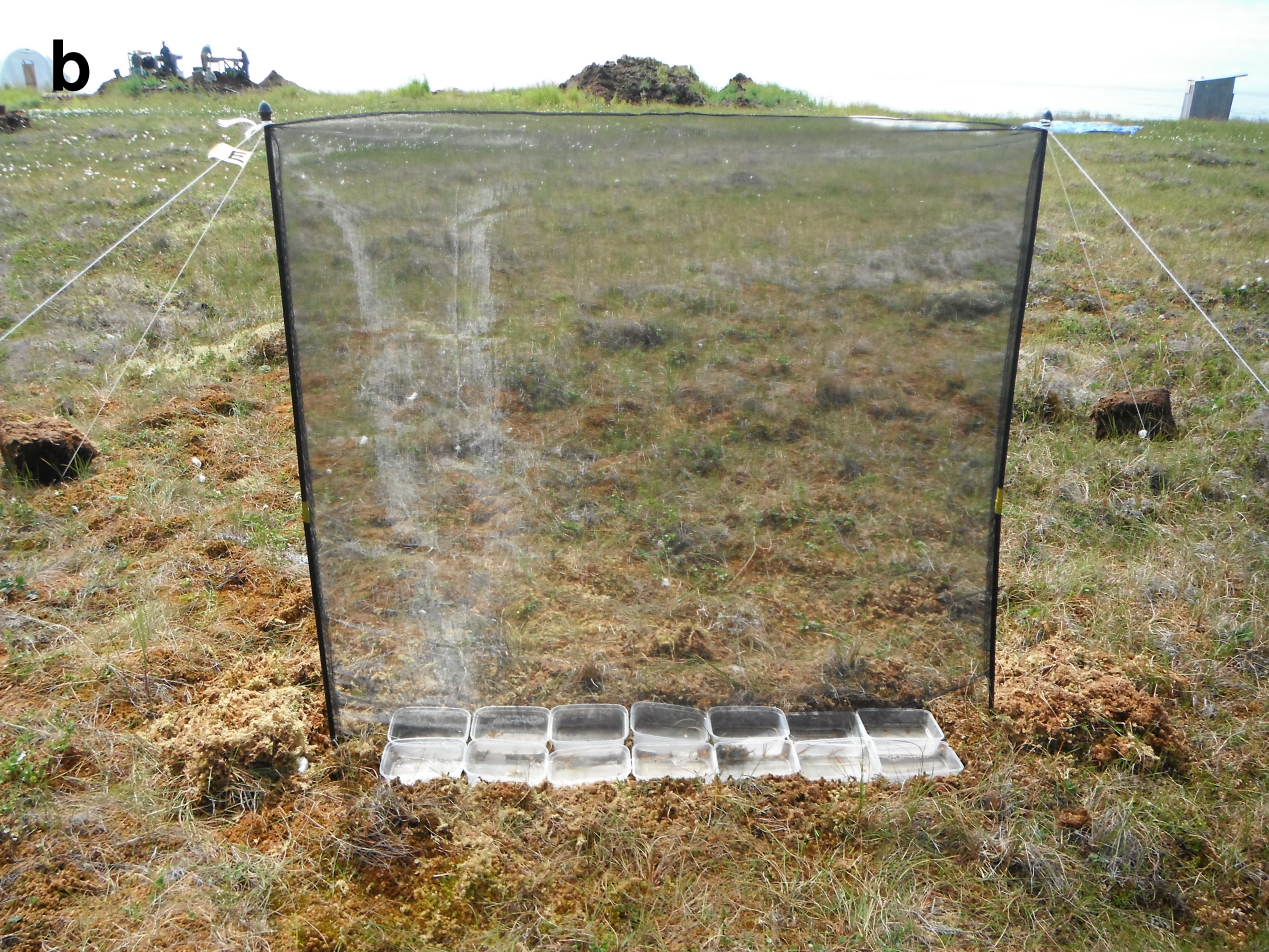
interception trap

**Figure 2c.**
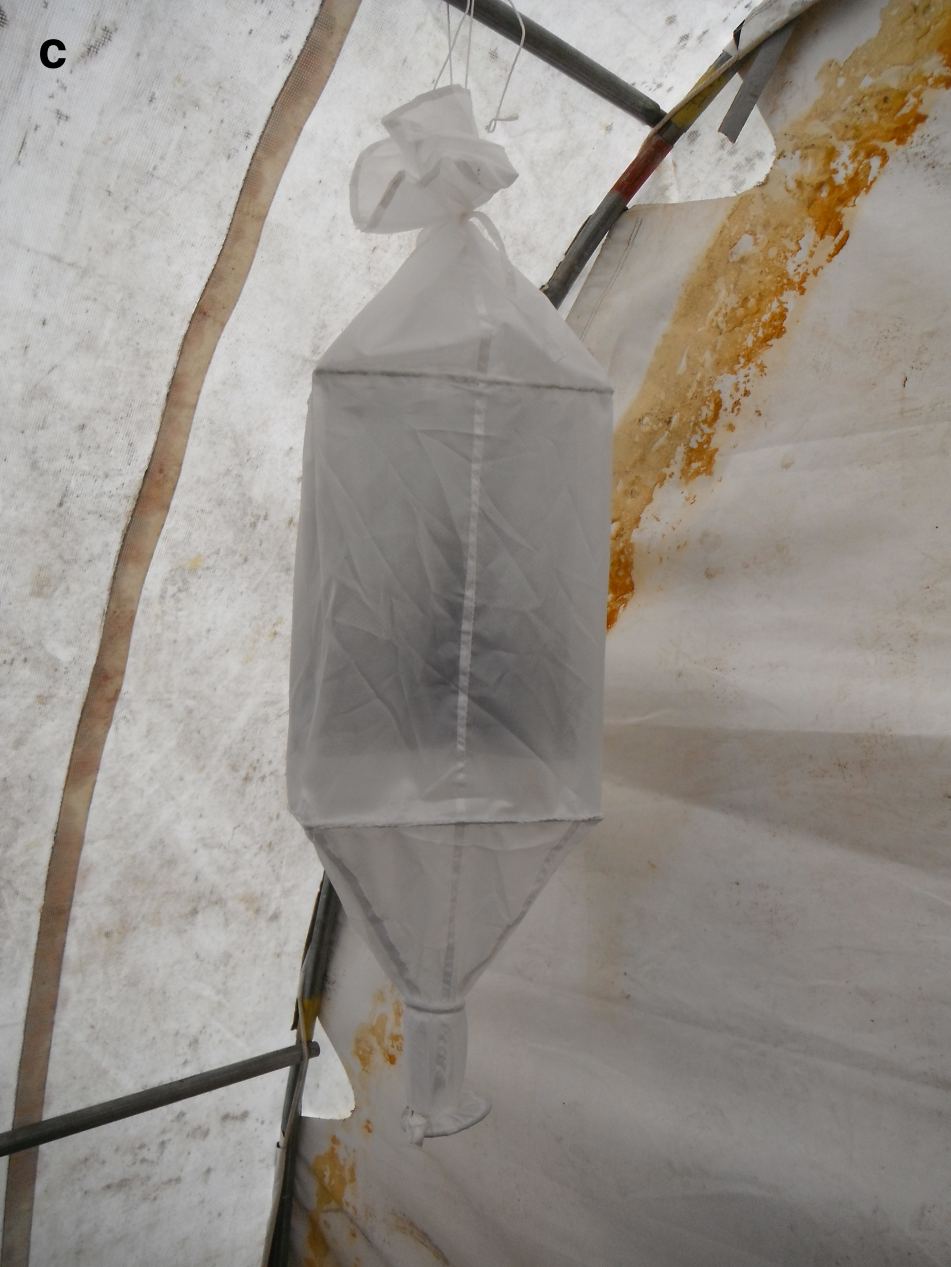
mini-Winkler extractor (supplied by Sante Traps http://www.santetraps.com/).

**Figure 3a.**
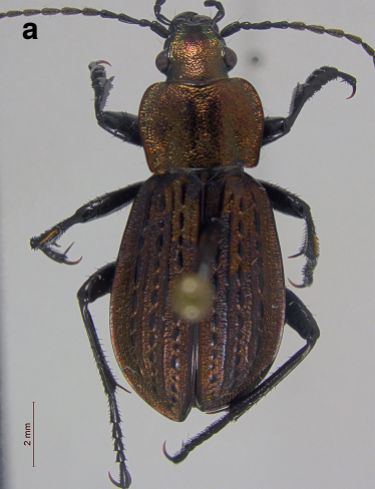
*Carabus maeander*

**Figure 3b.**
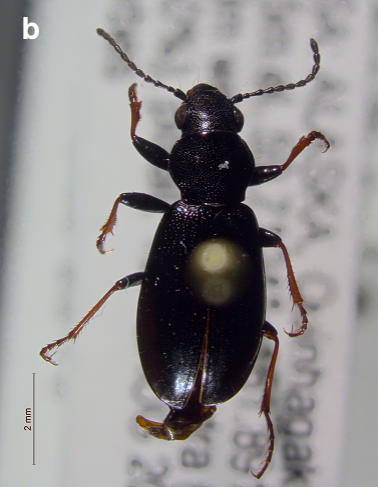
*Diacheila polita*

**Figure 3c.**
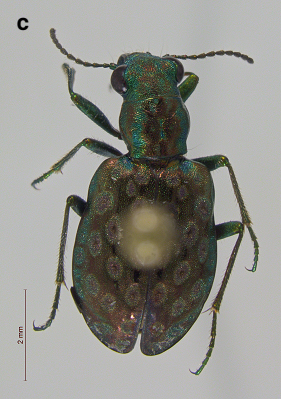
*Elaphrus trossulus*

**Figure 3d.**
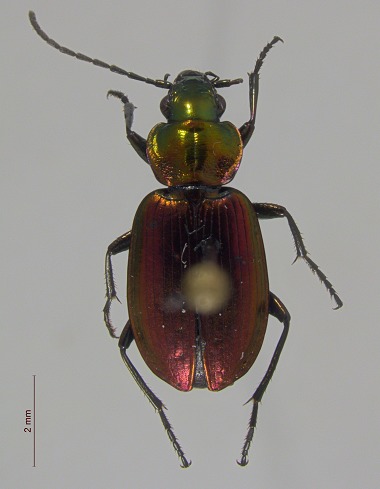
*Agonum quinquepunctatum*

**Figure 3e.**
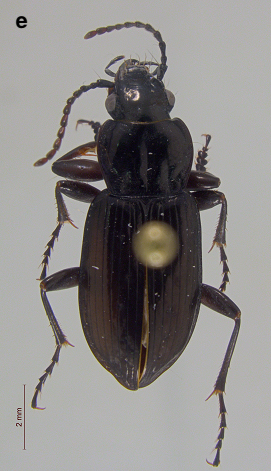
*Pterostichus adstrictus*

**Figure 3f.**
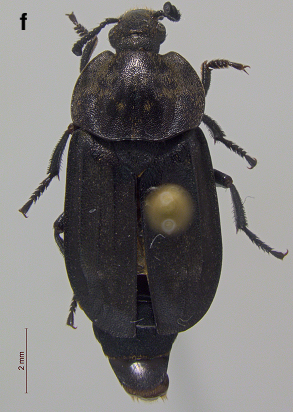
*Thanatophilus sagax*

**Figure 4a.**
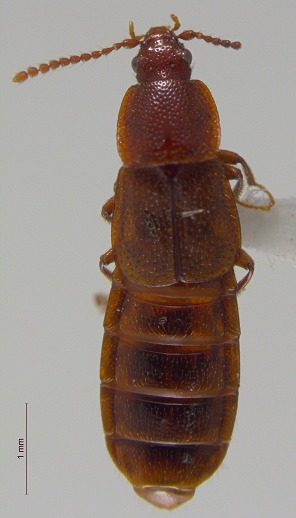
*Acidota quadrata*

**Figure 4b.**
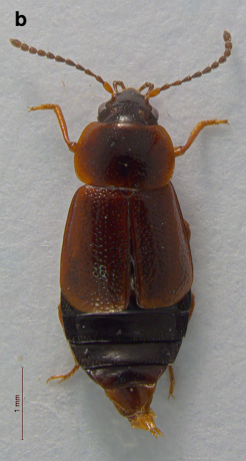
*Olophrum latum*

**Figure 4c.**
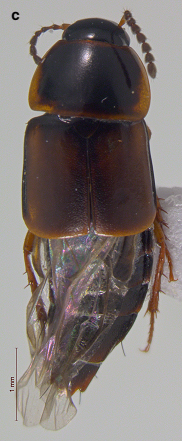
*Tachinus frigidus*

**Figure 4d.**
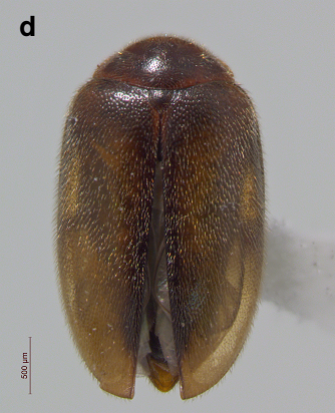
*Cyphon variabilis*

**Figure 4e.**
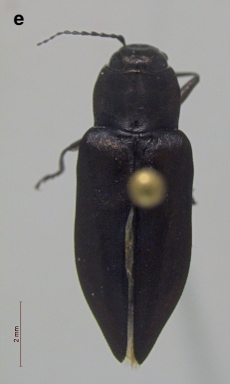
*Melanophila acuminata*

**Figure 4f.**
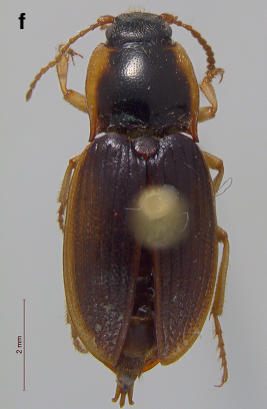
*Hypolithus littoralis*

**Table 1.** List and description of the different habitats sampled in the vicinity of the Nunalleq archaeological site.

**Habitat**	**Description**	**Sampling techniques**
Open tundra	Flat tundra with moist to wet ground and low-lying vegetation characterised by herbs (e.g. *Eriophorum angustifolium*), mosses (e.g. *Sphagnum* sp., *Polytrichium* sp.), lichen, heaths and dwarf shrubs (e.g. *Ledum palustre, Rubus chamaemorus, Empetrum nigrum, Betula nana*).	Pitfall and interception traps, hand collection, sifting/mini-Winkler
Scrub tundra	Flat tundra with moist to dry ground and vegetation dominated by dwarf willow (*Salix* sp.) scrub heaths and shrub (e.g. *Empetrum nigrum, Vaccinium vitis-idea, Betula nana, Rubus chamaemorus*).	Pitfall traps, beating vegetation
Aquatic	Small ponds of stagnant water with *Sphagnum* mosses and *Eriophorum angustifolium* at the water edge.	Dipping net and hand collection
Seashore	Beach with sandy and clayey soil, some areas with sparse vegetation (e.g. *Honckenya peploides, Senecio pseudoarnica, Mertensia maritima, Leymus arenarius*).	Pitfall traps, hand collection
Disturbed/anthropogenic	Habitats created through disturbance by human activity including spoil heaps, trampled areas and excavation trench of the Nunalleq archaeological site. Vegetation cover is typically sparse and characterised by, but not limited to, species such as *Achillea millefolium*, *Matricaria matricarioides*, *Rumex graminifolius* and *Rorippa islandica*. This category also includes the interior of modern buildings in Quinhagak.	Pitfall traps, hand collection
Riparian	Gravelly bank of the Arolik river, with willow (*Salix*) trees a few metres away from the water.	Beating vegetation, dipping net
